# Supplementary material for: Microbiological Evaluation of the Disinfecting Potential of UV-C and UV-C Plus Ozone Generating Robots
Source: Microorganisms. 2021 Jan 15;9(1):172. doi: 10.3390/microorganisms9010172 (PMC7830970; doi:10.3390/microorganisms9010172)

**Supplementary File S1.** Video film shot during the artificial surface contamination procedure and disinfecting action of robot 1.

**
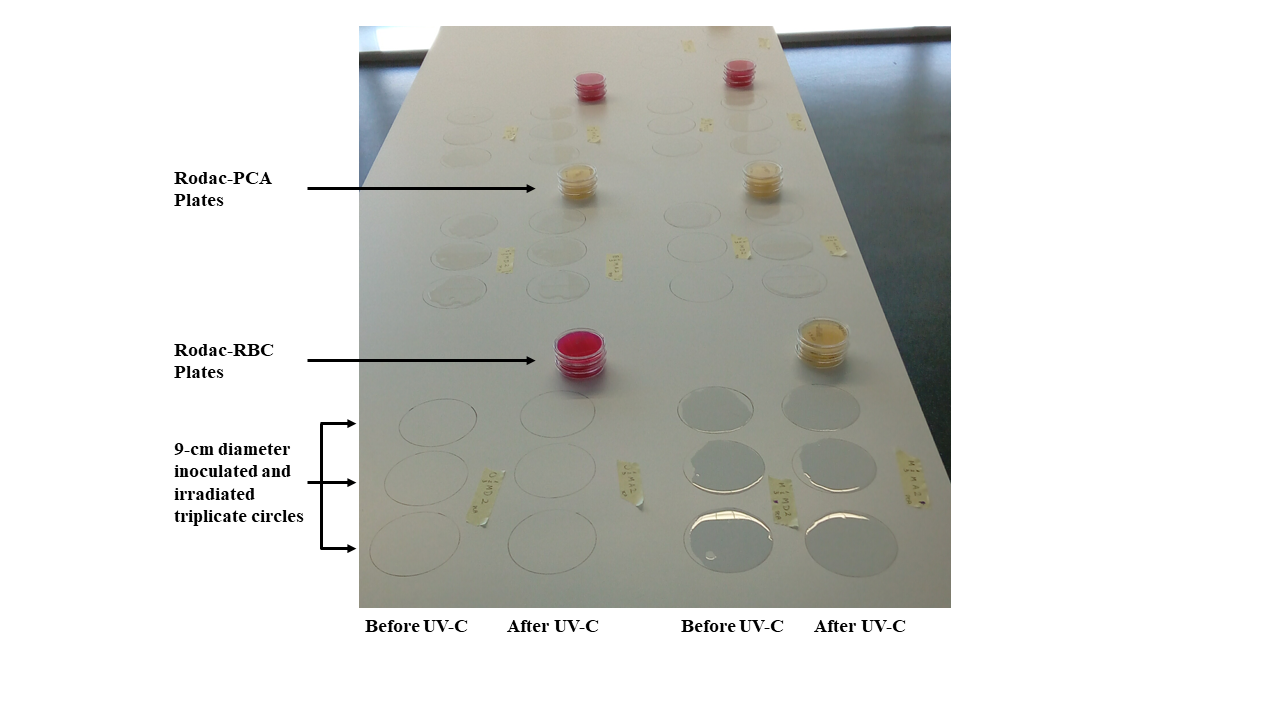
**

**Supplementary File S2.** Plate Count Agar (PCA) and Rose Bengal chloramphenicol (RBC) Rodac plates used to count bacteria and filamentous fungi and yeasts, respectively, are shown. Set of 9 cm diameter circles, three for N0 (before UV-C irradiation) and three for N (after UV-C irradiation) samples, marked on the table surface served to determinate the application area of each microbial suspension and the exact points where the 6.6 cm diameter Rodac plates had to be pressed onto the surface for 5 s for microorganism’s recovery.

**Supplementary File S3.** Bacteria number reduction for the robot 1 (40W, UV-C_245-256-nm_). Counting values, mean ± standard deviation (SD), and population reduction (%) of *E. coli*, *M. luteus*, and *B. subtilis* bacteria in Rodac-PCA plates, before and after disinfection treatment with the robot 1, on glass, floor, table, and wall surfaces.


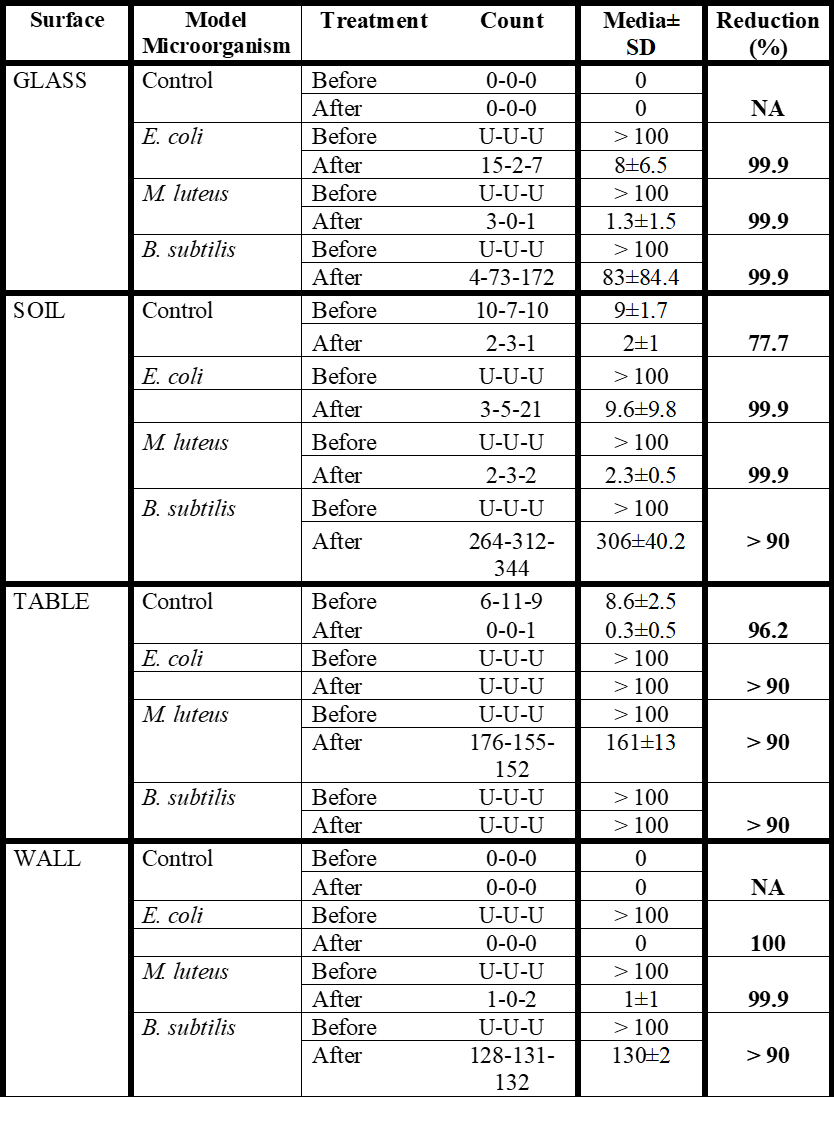


**Supplementary File S4.** Fungi number reduction for the Robot 1 (40W, UV-C_245-256-nm_). Counting values, mean ± standard deviation (SD), and population reduction (%) of *S. cerevisiae* and *T. harzianum* fungi in Rodac-BRC plates, before and after disinfection treatment with the robot 1, on glass, floor, table, and wall surfaces.


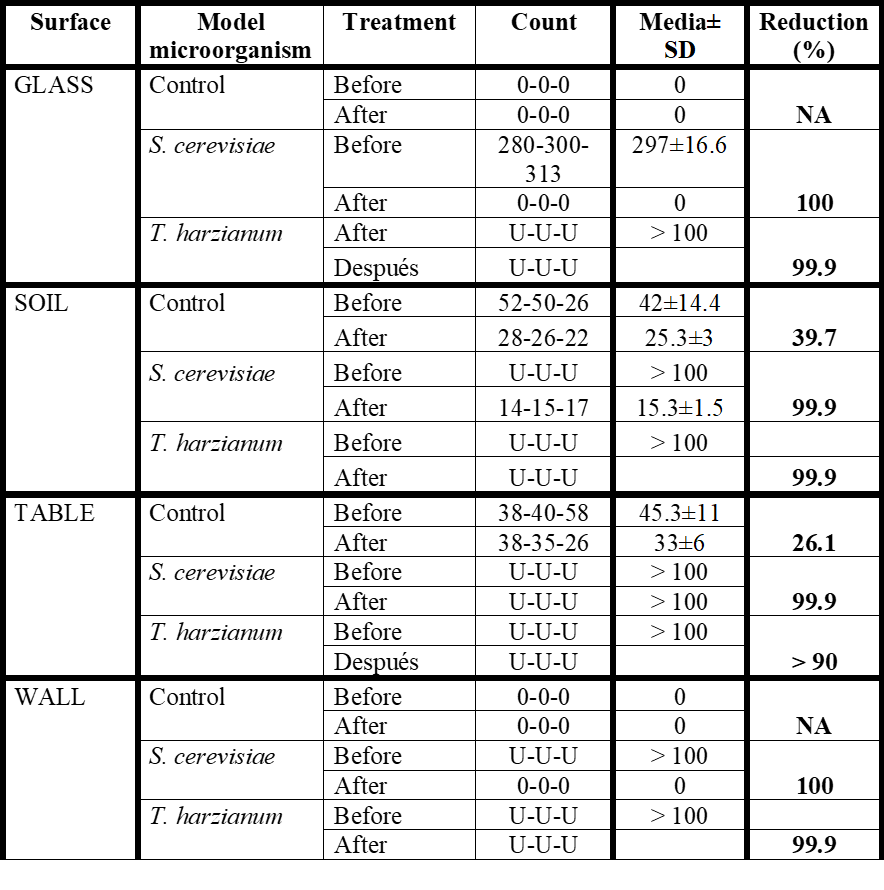


**Supplementary File S5.** Bacteria number reduction for the robot 2 (450 W, UV-C_185-254-nm_). Counting values, mean ± standard deviation (SD), and population reduction (%) of *E. coli*, *M. luteus*, and *B. subtilis* bacteria in Rodac-PCA plates, before and after disinfection treatment with ozone-generating robot 2, on glass, floor, table, and wall surfaces.

**
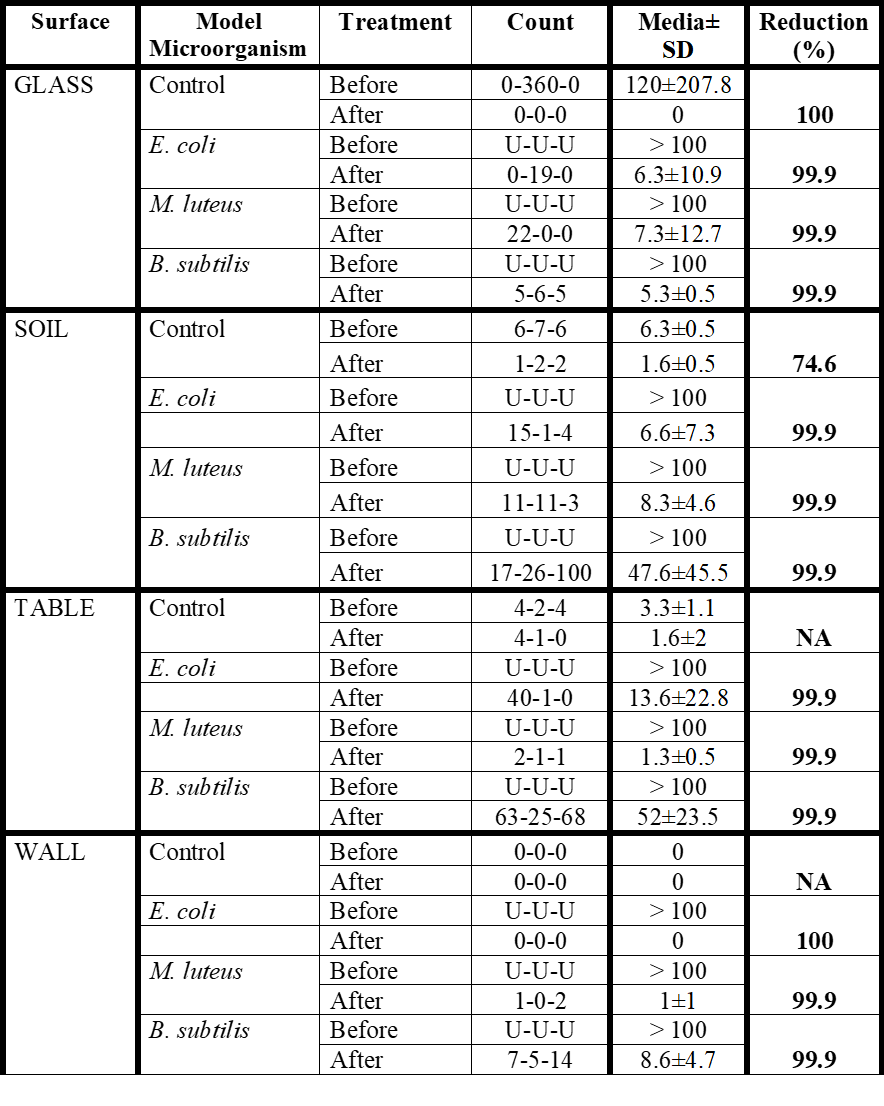
**

**Supplementary File S6.** Fungi number reduction for the robot 2 (450 W, UV-C_185-254-nm_). Counting values, mean ± standard deviation (SD), and population reduction (%) of *S. cerevisiae* and *T. harzianum* fungi in Rodac-BCR plates, before and after disinfection treatment with ozone-generating robot 2, on glass, floor, table, and wall surfaces.


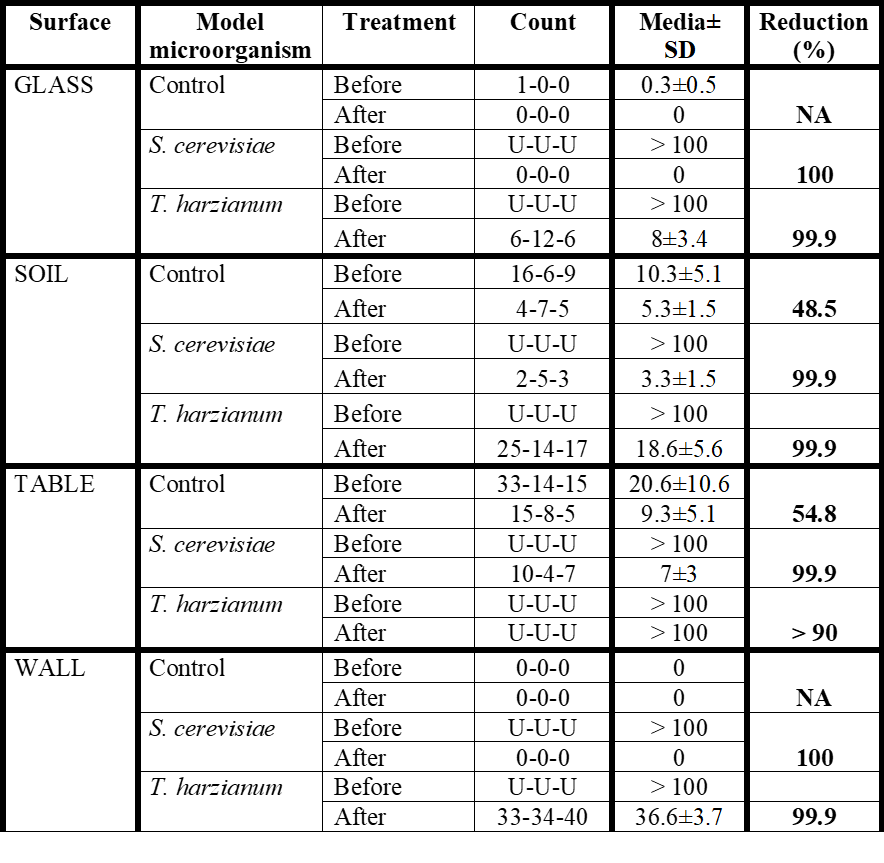

Supplement: Supplementary file 1 [file microorganisms-09-00172-s001.zip › microorganisms-1027538-sl-proofreading/microorganisms-1027538-sl-proofreading - cc.docx]
